# The role of CYP3A5 polymorphism and dose adjustments following conversion of twice-daily to once-daily tacrolimus in renal transplant recipients

**DOI:** 10.1186/s13737-016-0031-6

**Published:** 2016-01-28

**Authors:** Alina S. R Zaltzman, Lauren A. Glick, Jeffrey S. Zaltzman, Michelle Nash, Michael Huang, G. V. Ramesh Prasad

**Affiliations:** Faculty of Medicine, University of Ottawa, Ottawa, Canada; Faculty of Medicine, University of Toronto, Toronto, Canada; Division of Nephology and Transplant, Department of Medicine, St. Michael’s Hospital, Li-Ka Shing Knowledge Institute, University of Toronto, Toronto, Canada; Renal Transplant Program, St. Michael’s Hospital, Toronto, Canada

## Abstract

**Background:**

Tacrolimus is available as twice-daily Prograf® (Tac-BID) and the once-daily formulation, Advagraf® (Tac-OD). Although therapeutically equivalent, some transplant recipients require dose adjustments to achieve similar tacrolimus trough concentrations [Tac C_0_] after conversion between formulations. Tacrolimus is primarily metabolized by cytochrome *P450 3A5* (*CYP3A5*). We sought to determine whether genetic polymorphisms in the *CYP3A5* enzyme; CYP3A5 *1/*1 and CYP3A5 *1/*3 (expressers) compared to CYP3A5 *3/*3 (non-expressers) could account for discrepancies in dose requirements following conversion from Tac-BID to Tac-OD.

**Methods:**

A cohort of 60 renal transplant recipients (RTR) from our larger conversion study of 496 patients underwent additional testing for *CY3A5* genetic polymorphisms. Analysis included demographics, tac dosing and [Tac C_0_] pre- and post-conversion and dosing changes relative to *CYP3A5* genotypes. *CYP3A5* genetic polymorphisms were identified through analysis of genomic DNA.

**Results:**

Conversion from tac bid to tac OD in this cohort required a mean (SD) dose increase from 3.1 (1.0) mg/day to 3.8 (1.3) mg/day (*p* = 0.007), to achieve similar [Tac C_0_]. The *1/*3 expresser group required a greater percentage dose adjustment (56.7 %) in converting from Tac-BID to Tac-OD as compared to the *3/*3 non-expresser group (26.6 %). Similar findings were observed with the both expresser groups combined (*1/*1 &*1/*3). The expressers were significantly more highly represented in the East Asian cohort.

**Conclusions:**

The *CYP3A5* expresser polymorphism necessitates an increase in dosing upon conversion from Tac-BID to Tac-OD, with the expresser genotypes contributing significantly to this finding. Given the variability in frequency of *CYP3A5* genotypes in various ethnic groups, future studies should account for both isoenzyme polymorphism and ethnicity in optimizing dosing requirements.

**Trial registration:**

Clinical trials.gov identifier: NCT01884480

## Introduction

The calcineurin inhibitor tacrolimus is the most widely used immunosuppressive agent for the prevention of rejection following kidney transplantation. The branded agent is commercially available in two oral formulations, twice-daily Prograf® (Tac-BID) and once-daily Advagraf® or Astagraf® in the US (Tac-OD). Previous studies have shown the two formulations to be therapeutically equivalent [1, 2]. However, there is mounting evidence that a 1:1 conversion does not result in bioequivalence for all patients, with conversion from the BID to the OD formulation often resulting in under exposure as defined by tacrolimus trough concentration [Tac C_0_] [3–5].

Our previous work demonstrated that differences in ethnicity play a role in dose adjustment requirements when switching from Tac-BID to Tac-OD. In our Conversion Study of 496 renal transplant recipients (RTR) in a single centre converted from Tac-BID to OD, we found that 27.5 % of East Asians versus 13.5 % of Caucasians required a dose increase of 30 % or greater when switching from BID to OD [6].

*CYP3A5* has been identified as the major intestinal and hepatic enzyme responsible for Tac metabolism [7]. Variability in its pharmacokinetics has been explained by a single nucleotide polymorphism. The *CYP3A5* activity is largely determined by the single nucleotide variant (SNV) CYP3A5*3 (6986A > G; rs776746), which results in alternate mRNA splicing and a truncated and non-functional protein [8]. In homozygous carriers CYP3A5*3/*3 (non-expressers), the result is the absence of protein activity compared to the CYP3A5*1/*1 and CYP3A5*1/*3 (expressers) [9–11]. Non-expressers are believed to have a higher bioavailability and exposure of Tac, in part because of increased intestinal absorption and thus require lower doses to achieve target concentration compared to expressers [12].

Given that 20–30 % of patients may require some dose increase upon conversion from Tac-BID to Tac-OD to maintain similar trough tacrolimus concentrations [5, 6], we sought to explore whether genetic polymorphisms in the *CYP3A5* enzyme, in particular the CYP3A5*1/*1 and CYP3A5*1/*3 (expressers) compared to CYP3A5*3/*3 (non-expressers) could account for discrepancies in dose requirements following conversion from Tac-BID to Tac-OD.

## Materials and methods

As reported in our Conversion Study, 496 stable RTRs receiving Tac-BID were converted to Tac-OD [6]. In the large conversion trial, we examined dosing increases and decreases in the entire cohort required to maintain steady state [Tac C_0_] at 12 months post-conversion.

The current study involved recruitment of a cohort of 60 participants from the initial cohort of 496 with an aim to recruit recipients with a range of tac dosing requirements following conversion. We attempted to match based on age, transplant duration, renal function and transplant source. All [Tac C_0_] were done at our institution using high performance liquid chromatography mass spectrometer (HPLC-MS; Shimadzu Scientific Tokyo, Japan). Tacrolimus assay performance was characterized by six standardization references on a twice-daily basis. Genomic DNA obtained from whole blood using the MagNA Pure Compact Nucleic Acid Isolation Kit I (Roche Diagnostics, Laval, Quebec, Canada) was used to identify the variants of the cytochrome P450 enzyme *CYP3A5* (CYP3A5*3*3, CYP3A5*1*3, CYP3A5*1*1). The CYP3A5*3 (*g.6986A > G*, rs776746) TaqMan allelic discrimination assay (Life Technologies, Burlington, Ontario, Canada) was used for genotyping. Hardy–Weinburg equilibrium was assessed for genotypes using the *χ*^2^ goodness-of-fit test. SAS® software version 9.2 (Cary, NC, USA) was used for all analyses. Comparisons of tac dosing changes were done by paired *t* test and ANOVA for multiple comparisons. Chi-square analysis and Fisher’s exact tests were used for tac dose changes by genotype and genotyping by ethnicity. All participants provided written consent and the Research Ethics Board at St. Michael’s Hospital approved the study.

## Results

**Fig. 1:**
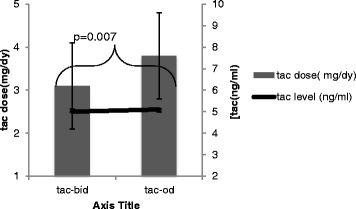
Tacrolimus dosing (mg/day) and mean tacrolimus trough concentration (ng/ml) pre- and post-conversion

Demographics of the study cohort are shown in Table 1. The study population was 46.7 % females, with 53 % having received living donor kidneys. The majority of recipients were Caucasian (53 %), 26.7 % were of East Asian origin and 8.3 % of South Asian descent. Recipients had been transplanted for a mean of 7.4 years prior to conversion, with an eGFR of 62 ml/min. As demonstrated in Fig. 1, upon conversion from Tac-BID to Tac-OD, tac dosing increased from 3.1 ± 1.0 mg/day to 3.8 ± 1.3 mg/day to maintain similar [tacC_0_] at 12 months post-conversion (*p* = 0.007). The mean haemoglobin was 132 ± 34 g/l. Fifteen patients were on diltiazem; however, there is no change in dose or frequency of use pre- or post-conversion.

**Table 1:** Demographics of transplant cohort

Variable	*N* = 60
Female (%)	28 (46.7)
Deceased donor (%)	32 (53.3)
Ethnicity (%)	
-Caucasian	34 (57)
-East Asian	16 (26.7)
-South Asian	5 (8.3)
-Black	2 (3.3)
-Other	3 (5.0)
Age at conversion	53.9 ± 9.5
Years post-transplant	7.4 ± 3.8
eGFR at conversion (ml/min)	62 ± 36.2
Tac BID dose pre-conversion (mg/day)	3.1 ± 1.0
Tac OD dose 12 months post-conversion (mg/day)	3.8 ± 1.3*
[Tac C_0_ BID] pre-conversion (ng/ml)	5.2 ± 1.6
[Tac C_O_ OD] post-conversion (ng/ml)	5.1 ± 1.5

*BID* twice daily, *OD* once daily, *Tac* tacrolimus

*p = 0.007 for Tac dose BID versus OD

### Genotypes

Of the cohort of 60, 43 (71.6 %) recipients were CYP3A5 *3/*3 (non-expressers), while 13 (21.7 %) were *1/*3 and 4 (6.7 %) *1/*1 (heterozygote and homozygote expressers, respectively).

### BID dosing (pre-conversion)

**Fig. 2:**
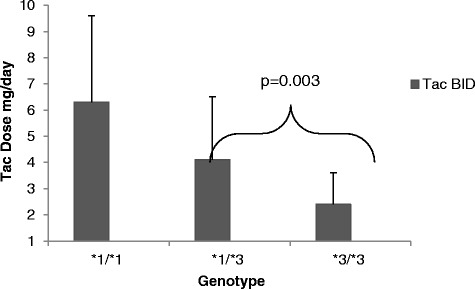
Tacrolimus dosing (mg/day) pre-conversion (Tac-BID) by genotype

As shown in Fig. 2, in comparing *CYP3A5* genotype groups, the *1/*1 expressers required the highest Tac-BID doses (6.3 ± 3.3), followed by the *1/*3 expressers (4.1 ± 2.4) and the *3/*3 non-expressers (2.4 ± 1.2). However, the *1/*1 group had a limited sample size of *n* = 4, and thus, no significant differences were shown with this group compared to the *1/*3 and *3/*3. There was a significant dose difference between the *1/*3 and *3/*3, *p* = 0.003.

### OD-dosing (12 months post-conversion)

**Fig. 3:**
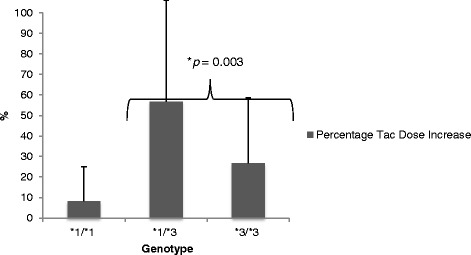
Percentage tacrolimus dose increase after conversion (Tac-OD) by genotype

Sixty-nine percent of the *1/*1 and *1/*3 (expressers) and 47 % of the *3/*3 (non-expressers) required some increase in dose following conversion (*p* = 0.004). The mean dose increase for the blended expresser cohort was 45.3 % (±48) versus 26.6 % (±32.1) for the non-expressers, *p* = 0.003. These results are highlighted in Table 2. As shown in Fig. 3, significant differences in dose increases following conversion were found for the *1/*3 (56.7 %) as compared to the *3/*3 (26.6 %). The mean dose increase for the four patients who expressed the *1/*1 genotype was 8.3 %.

**Table 2:** Tacrolimus dosing pre- and 12 months post-conversion comparison by genotype: expressers (*1/*1 + *1/*3) and non-expressers (*3/*3)

Variable	*1/*1 and *1/*3	*3/*3	*p* value
	*N* = 17	*N* = 43	
Required Tac dose increase (%)	10 (59)	20 (47)	0.004
Tac BID (mg/day)	4.6 ± 2.7	2.4 ± 1.2	0.0003
Tac OD (mg/day)	6 ± 2.7	2.9 ± 1.2	0.0001
Tac dose adjustment from BID to OD (%)	45.3 ± 48.3	26.6 ± 32.1	0.003

*BID* twice daily, *OD* once daily, *Tac* tacrolimus

*1/*3 expressers; *3/*3 non-expressers

### Ethnicity and genotype

As shown in Table 3, 8 recipients of 16 (50 %) of the East Asian cohort had *CYP3A5* expresser genotypes. This was significantly higher than the Caucasian cohort alone (*p* = 0.011). Of the East Asian cohort who were expressers, the mean dose increase following conversion was 53 % compared to 35 % increase for non-expressers. Owing to the small sample size, this result was not statistically significant.

**Table 3:** Genotype expression and ethnicity: expressers (*1/*1 + *1/*3) and non-expressers (*3/*3)

Ethnicity	Expressers (*1/*1 + *1/*3)	Non-expressers (*3/*3)
Caucasian	4 (13 %)	30 (87 %)
East Asian	7 (44 %)	9 (66 %)*
South Asian	2 (40 %)	3 (60 %)
Black	2 (100 %)	0 (0 %)
Other	2 (66 %)	1 (33 %)

*p = 0.011 for East Asians versus Caucasians

## Discussion

Initially, the recommended conversion from Tac-BID to Tac-OD was 1 mg:1 mg, as these were considered therapeutically equivalent [1, 2]. However, further experience has shown that tacrolimus exposure is reduced in some RTRs following conversion [3, 5, 6, 11]. The multicentre European conversion study of 1832 RTRs mandated an increase ratio of 1 mg of Tac BID to 1.1 mg of Tac OD in participants with [Tac C_0_] < 6 ng/ml [13]. In our cohort of 60, a mean tac increase of 18 % post-conversion was required to achieve steady state [Tac C_0_] by 12 months. Our study suggests that dose increase upon conversion may be directly related to differences in *CYP3A5* polymorphisms. In particular, the highest increase in Tac dose following conversion was seen in *CYP3A5* expressers. In comparing the genotypes, the *1/*1 expressers required the highest Tac-BID and Tac-OD doses, followed by the *1/*3 expressers and the *3/*3 non-expressers. This is consistent with previous research on both adult and paediatric populations, reporting that expressers require higher doses of tacrolimus, due to higher oral clearance [14, 15].

It was demonstrated that the *1/*3 expressers and the combined *1/*3 and *1/*1 expressers required significantly higher doses of Tac-OD after conversion from Tac-BID. The increase in dose requirement upon conversion was attributed primarily to the *1/*3 expresser cohort because while the homozygous*1/*1 represents the greatest “expression”, this cohort was a relatively small sample (only 4/60) and therefore underpowered as a single group to contribute meaningfully to the data. Similar findings were demonstrated by Glowacki et al. who showed that RTRs with the expresser genotype required a dose increase upon conversion compared to no increase in non-expresser recipients [16].

There are inconsistent reports on expresser phenotypes and Tac-OD, with Niioka demonstrating reduced Tac exposure in *1/*1 and *3/*3 recipients [17]. Some groups have found opposing effects of *CYP3A5* polymorphisms on the pharmacokinetics of Tac-OD, with *3/*3 patients demonstrating significantly lower tacrolimus concentration and area under the curve after conversion, with no change in levels for the *1/*1 patients [18]. These results have been compatible with previous research in the paediatric population [3, 19]. However, the paediatric studies had relatively small sample sizes, with unequal or unclear distribution of *CYP3A5* polymorphisms, thereby making comparisons to the current study more difficult. Additionally, body weight has a significant impact on the pharmacokinetics of tacrolimus in the paediatric population, which was not accounted for in the current adult study.

Genetic polymorphisms influence the pharmacokinetic variability between individuals. Oral clearance of tacrolimus is higher in CYP3A5*1 expressers (lower dose-adjusted [C0]), as these individuals have the isoenzyme enhancing drug metabolism [10, 20]. Therefore, CYP3A5*3 patients have greater bioavailability of tacrolimus and require lower doses to achieve target blood levels [9, 10]. Niioka et al. reported that tacrolimus bioavailability was associated with both the *CYP3A5* polymorphism and the oral formulation (BID versus OD). Bioavailability was lowest in recipients with the CYP3A5*1 allele taking Tac-OD. The Tac-OD formulation is released more slowly, and thus absorbed, further along the GI tract and thus has longer gut exposure time than the Tac-BID formulation [17]. Since *CYP3A5* is a resident throughout the small intestine, greater exposure in expressers (*1/*1 &*1/*3) can result in even more metabolism of tacrolimus to its inactive metabolite desmethyl FK-506 [21] compared to *3/*3 non-expressers. A difference in CYP3A5 protein expression level in the small intestine may influence the oral bioavailability of tacrolimus in both initial exposure to Tac and in steady state. Therefore, *CYP3A5* expression has a greater influence on the pharmacokinetics of Tac-OD than Tac-BID.

In our larger study of 496 recipients converted from Tac-BID to Tac-OD, it was found that 16 % of RTRs required a dose increase of at least 30 % upon conversion to Tac-OD. Of these individuals, dose increases were required most frequently in the East Asian cohort [6]. CYP3A5*1/*1 and *1/*3 genotypes are believed to be more prevalent in East Asians, as compared to other ethnic groups such as Caucasians [22]. This current work further validates the data from our larger conversion trial. The 16 East Asian recipients had a 50 % “expresser genotype” which was statistically different from both the largest cohort of 34 Caucasians. Previous research examining the East Asian patient population has reported similar findings that *CYP3A5* expressers require higher doses of Tac-OD than Tac-BID. Niioka et al. reported 25 % lower area-under-the-curve values upon conversion to Tac-OD in Japanese RTRs [17]. Furthermore, this was primarily in those participants with the CYP3A5*1/*3 genotype. Additionally, Zhang et al. found that Chinese CYP3A5*1/*3 expressers required significant dose increases upon conversion in order to maintain therapeutic tacrolimus blood levels as compared to CYP3A5*3/*3 non-expressers [23]. The results of the present study confirm these findings, suggesting that *CYP3A5* genotype contributes significantly to tacrolimus requirements upon conversion, more specifically in the CYP3A5*1/*3 subtype. In the current study, the cohort size of Black patients was too small to examine variations in genotype in this cohort. However, it can be hypothesized that the higher dose requirements upon conversion in the *CYP3A5* expresser cohorts (*1/*1 and *1/*3) might occur with the greatest frequency in the East Asian population. However, ethnicity alone may not be predictive of dose increase. As evidenced by results from our larger study and others [24], the African American cohort required the highest doses of Tac both pre- and post-conversion. However, the required increase post-conversion was <1 % of the pre-conversion dose [6].

The small sample size of the *1/*1 was a limitation of our study. However, the homozygous expresser group in general is underrepresented in the renal transplant population. In addition, this study did not address other proteins involved in tacrolimus metabolism such as P-glycoprotein or *CYP3A4* [25, 26].

## Conclusions

The present study confirms that conversion from Tac-BID to Tac-OD necessitates an increase in dosage for RTRs with *CYP3A5* expresser polymorphism. The magnitude of this increase can be attributed to the heterozygous expresser genotype (*1/*3), and likely the homozygous *1/*1. Ideally, genetic polymorphism testing prior to tacrolimus dosing may help to achieve the target tacrolimus blood levels post-transplant. However, given that there appears to be a variation in the proportion of expresser genotypes between ethnic groups, ideal clinical application should account for both ethnicity and *CYP3A5* polymorphisms to optimize initial tacrolimus dosing and conversion strategies.

## Abbreviations

CYP3A5: cytochrome P450 3A5
RTR: renal transplant recipients
Tac: tacrolimus
Tac-BID: tacrolimus twice-daily
Tac-OD: tacrolimus once-daily
Tac C_0_: trough concentration
